# Age-related circadian disorganization caused by sympathetic dysfunction in peripheral clock regulation

**DOI:** 10.1038/npjamd.2016.30

**Published:** 2017-01-05

**Authors:** Yu Tahara, Yuta Takatsu, Takuya Shiraishi, Yosuke Kikuchi, Mayu Yamazaki, Hiroaki Motohashi, Aya Muto, Hiroyuki Sasaki, Atsushi Haraguchi, Daisuke Kuriki, Takahiro J Nakamura, Shigenobu Shibata

**Affiliations:** 1 Laboratory of Physiology and Pharmacology, School of Advanced Science and Engineering, Waseda University, Tokyo, Japan; 2 Waseda Institute for Advanced Study, Waseda University, Tokyo, Japan; 3 Laboratory of Animal Physiology, School of Agriculture, Meiji University, Kanagawa, Japan

## Abstract

The ability of the circadian clock to adapt to environmental changes is critical for maintaining homeostasis, preventing disease, and limiting the detrimental effects of aging. To date, little is known about age-related changes in the entrainment of peripheral clocks to external cues. We therefore evaluated the ability of the peripheral clocks of the kidney, liver, and submandibular gland to be entrained by external stimuli including light, food, stress, and exercise in young versus aged mice using *in vivo* bioluminescence monitoring. Despite a decline in locomotor activity, peripheral clocks in aged mice exhibited normal oscillation amplitudes under light–dark, constant darkness, and simulated jet lag conditions, with some abnormal phase alterations. However, age-related impairments were observed in peripheral clock entrainment to stress and exercise stimuli. Conversely, age-related enhancements were observed in peripheral clock entrainment to food stimuli and in the display of food anticipatory behaviors. Finally, we evaluated the hypothesis that deficits in sympathetic input from the central clock located in the suprachiasmatic nucleus of the hypothalamus were in part responsible for age-related differences in the entrainment. Aged animals showed an attenuated entrainment response to noradrenergic stimulation as well as decreased adrenergic receptor mRNA expression in target peripheral organs. Taken together, the present findings indicate that age-related circadian disorganization in entrainment to light, stress, and exercise is due to sympathetic dysfunctions in peripheral organs, while meal timing produces effective entrainment of aged peripheral circadian clocks.

## Introduction

Physiological events that show day-night fluctuations in mammals are controlled by an internal circadian clock system. The suprachiasmatic nucleus (SCN) of the hypothalamus is thought to be the master pacemaker of this system.^1–3^ Within recent decades, a feedback loop of clock genes known as the molecular clock was identified in the cells of all tissues; moreover, it was found that the SCN provides regulatory input these peripheral clocks.^1,3^ In mice, the mutation or deletion of clock genes leads to the disruption of homeostasis and the accumulation of reactive oxygen species, which in turn can result in the development of diseases including metabolic syndrome, cancer, and cardiovascular disease.^1,4–7^ Conversely, many diseases including obesity, cancer, and dementia have been reported to attenuate the circadian clock system in mice and humans.^1,8–10^

In humans, the process of aging is associated with a decline in diurnal variations in the sleep–wake cycle, body temperature and hormonal secretions.^11–13^ Age-associated decline of the timing signal from the SCN has been reported to manifest in the neural firing rhythms and membrane properties of SCN cells.^13,14^ In addition, the molecular clock was normally oscillated in the aged SCN *in vivo,* but faster decline of oscillation was seen in cultured SCN from aged mice than that from young mice.^14–20^ A small number of studies have measured molecular clock oscillations in aged peripheral tissues.^15,17,18,21,22^ Some of these studies have employed luciferase reporter transgenic mice or rats to compare core clock gene (*Per1* and *Per2)* expression in liver, lung, pineal gland, and kidney tissues from young and old animals.^17,18,21^ Although aged peripheral clocks typically exhibit normal oscillatory patterns, experimental jet lag induced by shifting of the light–dark cycle has been reported to produce abnormal phase changes in aged but not young mice.^17,18,21^ Furthermore, chronic jet lag exposure increases the mortality rate in aged mice.^23^ Despite these apparent relationships between circadian rhythm and peripheral clock function in aged mice, no study to date has examined changes in the coordination of the SCN with peripheral clocks in aging.

Entrainment or phase adaptation is an important function of the circadian clock system that allows the adjustment of circadian dynamics in response to external stimuli. Peripheral clock entrainment is internally mediated by the SCN in response to nervous, endocrine and feeding behavioral inputs.^1,3^ Accordingly, the circadian system can be entrained by external cues such as light, food, stress, and exercise.^1–3,24^ Studies indicate that peripheral clocks are more efficiently entrained by food, stress, and exercise stimuli than light–dark stimuli.^2,3,25^ Therefore, to understand the properties of the circadian system in aged mice, we compared the ability of peripheral circadian clocks to be entrained by external stimuli in young and aged mice. We recently established the utility of *in vivo* whole-body imaging of peripheral PER2::LUCIFERASE (PER2::LUC) bioluminescence for the monitoring of peripheral clock phases.^26^ In the present study, this method enabled us to visualize individual peripheral clocks in a non-invasive and longitudinal manner that is further compatible with existing aging research.

## Results

### Peripheral clocks in aged mice exhibit normal oscillations under light–dark, constant darkness, and stimulated jet lag conditions

**Figure 1:**
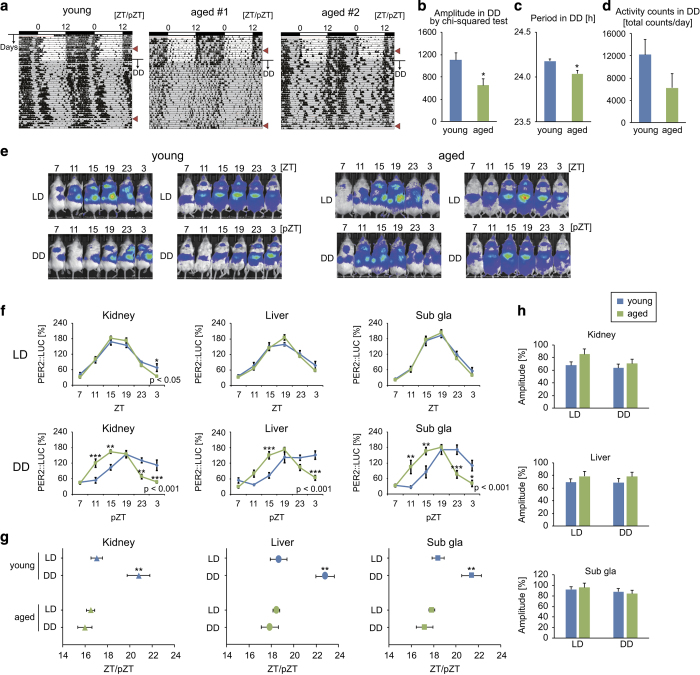
Locomotor activity and peripheral PER2::LUC rhythms under light–dark and constant darkness conditions. (**a**) Representative double-plotted actograms of locomotor activity measured by an infrared sensor in young (*n*=1) and aged (*n*=2) mice maintained under normal light–dark (LD) or constant darkness (DD) conditions for 1 month. The dark shadow indicates the dark (active) period. Arrowheads on the right indicate the timing of *in vivo* monitoring of peripheral PER2::LUC bioluminescence. (**b**–**d**) Amplitude (**b**) and period (**c**) of the rhythmicity of locomotor activity analyzed by *χ*^2^ periodogram, and total daily activity (**d**) in the DD condition (*n*=4–5). (**e**–**h**) Representative photo images (**e**), analyzed wave forms (**f**), peak phases (**g**), and amplitudes (**h**) of PER2::LUC bioluminescence in the kidney, liver, and submandibular gland (sub gla) of young and aged mice in each condition. ZT, zeitgeber time; pZT, projected ZT in constant darkness (pZT0 and pZT12 representing the same times as ZT0 and ZT12, respectively). Values are expressed as the mean±s.e.m. The number of mice used is indicated in Supplementary Table S1. *P<0.05, **P<0.01, ***P<0.001 versus the young group or the LD group (Student’s *t* test or two-way analysis of variance (ANOVA) with Tukey’s or Sidak’s *post hoc* tests). The *P* value on the lower right side of each figure (**f**) indicates the result of two-way ANOVA between the young and aged groups.

**Figure 2:**
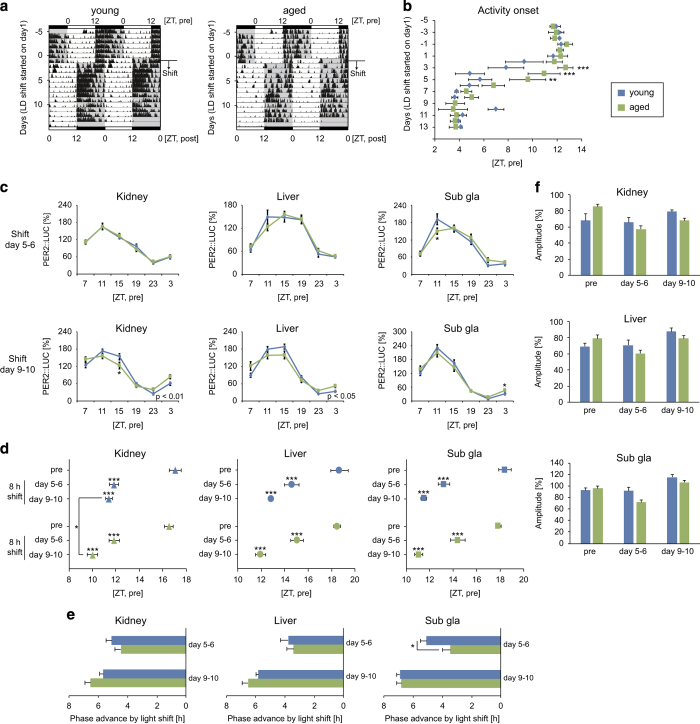
Locomotor activity and peripheral PER2::LUC rhythms under the experimental jet lag condition. (**a**) Representative double-plotted actograms of locomotor activity as measured by an infrared sensor in young and aged mice. The dark shadow indicates the dark period. (**b**) Averaged time of activity onset during the shifting phase of the light–dark cycle (*n*=8). (**c**–**e**) Analyzed wave forms (**c**), peak phases (**d**), phase advance values (**e**), and amplitudes (**f**) of PER2::LUC bioluminescence in the kidney, liver, and submandibular gland (sub gla) of young and aged mice 5 or 9 days after the light–dark cycle phase shift (different mouse cohorts were used on day 5 and day 9). Phase advance values were calculated as the difference between the peak times before and after the light–dark cycle phase shift. Values are expressed as the mean±s.e.m. The number of mice used is indicated in Supplementary Table S1. *P<0.05, **P<0.01, ***P<0.001 versus the young group or the pre group (Student’s *t* test, 1-way analysis of variance (ANOVA) with Dunnett *post hoc* tests, or two-way ANOVA with Sidak’s *post hoc* tests). The *P* value on the lower right side of each figure (**c**) indicates the result of a two-way ANOVA between the young and aged groups.

In the constant darkness condition, aged (>18 months) PER2::LUC ICR mice showed a lower amplitude of locomotor rhythmicity and a shorter period of free-running activity than young (3–6 months) female mice (Figure 1a–d). However, PER2::LUC bioluminescence rhythms in the kidney, liver, and submandibular gland showed clear cosine-like circadian rhythms in the normal light–dark and constant darkness conditions in both young and aged mice (Figure 1e–h, Supplementary Table S1). Similarly, no age-dependent differences were noted in peripheral PER2::LUC rhythms for male mice in the light–dark condition (Supplementary Figure S1B). A delay in the peak phases of peripheral clocks in young mice was observed in the constant darkness condition relative to the delayed phase of behavioral rhythms (Figure 1g). Similar to the observed PER2::LUC rhythms, mRNA expression of *Per1* and *Per2* in each tissue showed normal oscillations in both young and aged mice (Supplementary Figure S1). For the simulated jet lag condition, phase advance of the activity rhythm following an 8-h advance of the light–dark cycle was slower in aged mice than in young mice, as described previously;^21^ however, by day 5, slower peripheral clock phase changes were only detected in the submandibular gland (Figure 2a–e). By days 9 and 10, a faster phase advance was noted in the kidney in the aged group relative to the young group. Taken together, these data indicate that the peripheral clocks of aged mice are almost normal in the presence or absence of light–dark information, even if SCN-driven behavioral rhythms are impaired.

### Enhanced peripheral clock entrainment to feeding signals in aged mice

**Figure 3:**
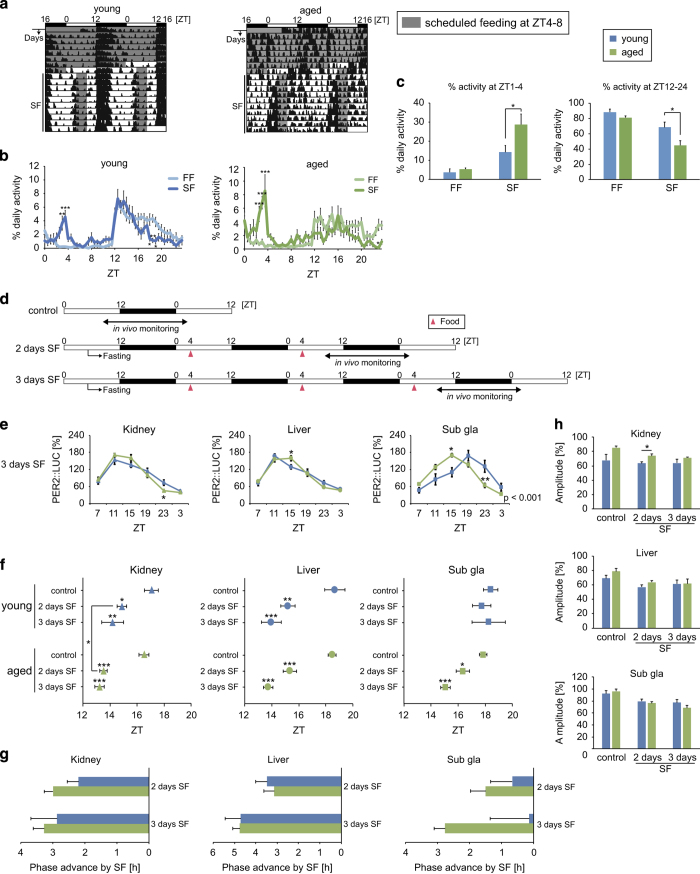
Locomotor activity and peripheral PER2::LUC rhythms in response to daytime scheduled feeding. (**a**) Representative double-plotted actograms of locomotor activity as measured by an infrared sensor in young and aged mice. The dark shadow indicates the feeding period. (**b**) Wave forms of locomotor activity analyzed during the last 3 days of each condition (*n*=8). FF, free feeding; SF, scheduled feeding. (**c**) Per cent activity during 3 h prior to the scheduled feeding time (Zeitgeber time (ZT) 1–3) or during the dark period (ZT12–24). (**d**) The experimental feeding schedule. White and black bars indicate the light and dark periods, respectively. Arrowheads indicate food timings. Food pellets (1 g for the first and second days, 1.5 g for the third day) were given to mice after overnight fasting. (**e**–**h**) Analyzed wave forms (**e**), peak phases (**f**), phase change values (**g**), and amplitudes (**h**) of PER2::LUC bioluminescence in the kidney, liver, and submandibular gland (sub gla) of young and aged mice in each condition. Values are expressed as the mean±s.e.m. The number of mice used is indicated in Supplementary Table S1. *P<0.05, **P<0.01, ***P<0.001 versus the young group or the control group (Student’s *t* test, 1-way analysis of variance (ANOVA) with Dunnett’s *post hoc* tests, or two-way ANOVA with Sidak’s *post hoc* tests). The *P* value on the lower right side of each figure (**e**) indicates the result of a two-way ANOVA between the young and aged groups.

To measure the ability of peripheral clocks to be entrained by feeding cues in young versus aged mice, food-induced phase entrainment was performed as described previously (Figure 3).^27,28^ Young and aged mice exhibited food anticipatory behaviors after 10 days of scheduled feeding; however, the ratio of food anticipatory activity was greater in aged mice than in young mice (Figure 3a–c). In young mice, 2 or 3 days of scheduled feeding-induced phase advances were observed in the kidney and liver but not in the submandibular gland (Figure 3d–h), which is consistent with previous reports.^29,30^ In aged mice, PER2::LUC rhythms in the liver and kidney showed similar phase entrainments to those in young mice; however, a phase-shift was also noted in the aged submandibular gland. Because of the use of female mice in the present study, we reproduced this experiment in ovariectomized young female mice (Supplementary Figure S2) and did not note any feeding schedule-induced phase-shifts in the submandibular gland of ovariectomized mice, suggesting that a lack of oestrus hormones was not involved in the observed age-related difference. Thus, aged-related differences were noted in the entrainment of food anticipatory activity and the peripheral clock of the submandibular gland to scheduled feeding.

**Figure 4:**
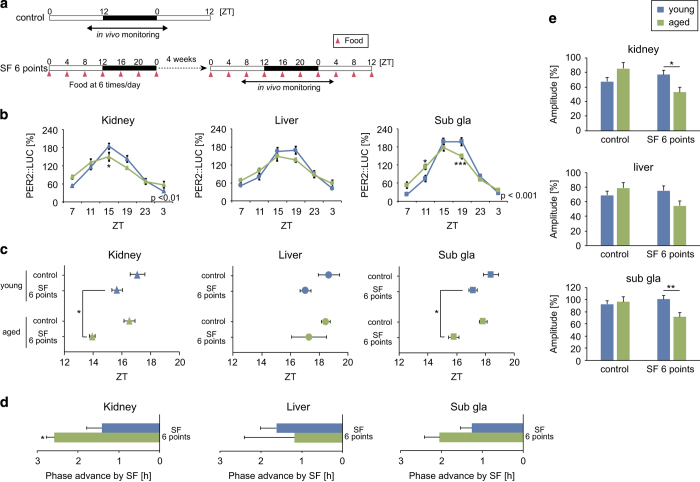
Effect of constant routine feeding on peripheral PER2::LUC rhythms. (**a**) Experimental constant routine feeding schedule. Mice were fed the same amount of food (0.54 g) 6 times per day at the same interval for 4 weeks. White and black bars indicate the light and dark periods, respectively. Arrowheads indicate food timings. ZT, zeitgeber time; SF, scheduled feeding. (**b**–**e**) Analyzed wave forms (**b**), peak phases (**c**), phase change values (**d**), and amplitudes (**e**) of PER2::LUC bioluminescence in the kidney, liver, and submandibular gland (sub gla). Values are expressed as the mean±s.e.m. The number of mice used is indicated in Supplementary Table S1. In evaluation of the aged kidney, 1 outlier was excluded. *P<0.05, **P<0.01 versus the young group (Student’s *t* test, Mann–Whitney test, or two-way analysis of variance (ANOVA) with Sidak’s or Tukey’s *post hoc* tests). The *P* value on the lower right side of each figure (**b**) indicates the result of a two-way ANOVA between the control and SF 6 points groups.

Because we observed the continued oscillation of peripheral circadian clocks in aged mice despite constant darkness (Figure 1), we hypothesized that the fasting-feeding cycle was more important than the light–dark cycle for maintaining circadian oscillations in peripheral organs. Therefore, we experimentally diminished circadian feeding cycle cues by providing scheduled feedings 6 times per day at equally spaced intervals (i.e., the constant routine experiment)^31^ for 1 month in young and aged mice (Figure 4a–e). In this condition, amplitude of the peripheral clocks were significantly dampened in aged mice relative to young mice, suggesting that the fasting-feeding cycle is important for the entrainment of peripheral clocks in aged mice.

### Attenuated peripheral clock entrainment to stress and exercise signals in aged mice

**Figure 5:**
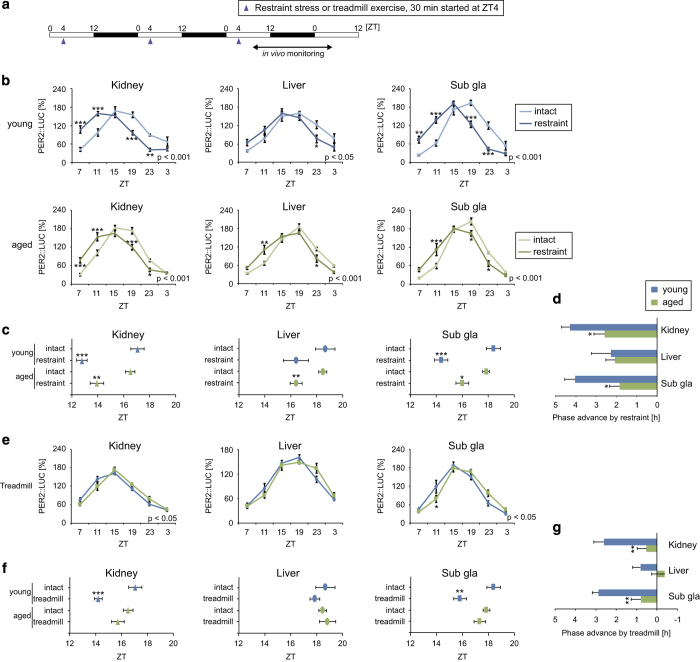
Peripheral PER2::LUC rhythms in response to daily scheduled restraint stress or treadmill exercise. (**a**) Experimental restraint stress and treadmill exercise schedules. White and black bars indicate the light and dark periods, respectively. Arrowheads indicate stimulation timings. ZT, zeitgeber time. (**b**–**g**) Analyzed wave forms (**b** and **e**), peak phases (**c** and **f**), and phase change values (**d**, **g**) of PER2::LUC bioluminescence in the kidney, liver, and submandibular gland (sub gla) of young and aged mice in each condition. Phase advance values were calculated as the difference between the peak times of the restraint/treadmill group and the intact group. Values are expressed as the mean±s.e.m. The number of mice used is indicated in Supplementary Table S1. *P<0.05, **P<0.01, ***P<0.001 versus the young group or the intact group (Student’s *t* test or two-way analysis of variance (ANOVA) with Sidak’s or Tukey’s *post hoc* tests). The *P* value on the lower right side of each figure (**b**, **e**) indicates the result of a two-way ANOVA on the intact and restraint groups or the young and aged groups.

Peripheral clock phase adjustment has also been reported in response to scheduled daytime restraint stress or treadmill exercise.^25,32,33^ Compared with young mice, aged mice showed significantly weaker entrainment responses to these stimuli in the kidney and submandibular gland (Figure 5a–g). In addition, serum corticosterone concentrations were significantly lower before and after restraint stress in aged mice than in young mice (Supplementary Figure S3). Conversely, mRNA expression of the glucocorticoid receptor in the submandibular gland was not significantly different between young and aged mice (Supplementary Figure S3, Supplementary Table S3). To determine whether differences in stress or exercise-induced glucocorticoid activation contributed to differences in the entrainment of peripheral clocks according to age,^32,33^ mice were treated daily with the glucocorticoid analogue dexamethasone (DEX) and a similar phase change was observed in the submandibular gland (Supplementary Figure S3). Furthermore, the phase advancement in response to DEX was smaller in aged mice than in young mice. These results suggest that an age-related decline in glucocorticoid regulation may mediate impaired peripheral circadian clock entrainment in response to stress/exercise stimuli.

### Attenuated sympathetic regulation of the submandibular gland in aged mice

**Figure 6:**
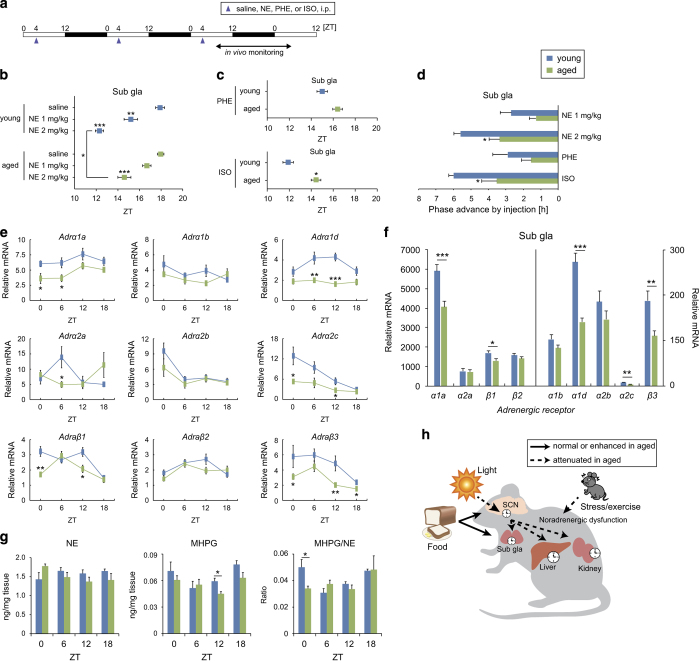
Age-related changes in sympathetic regulation of the submandibular gland. (**a**) Experimental schedule of adrenergic receptor stimulation. Saline (vehicle), norepinephrine (NE), an α-adrenergic receptor agonist (phenylephrine; PHE), and a β-adrenergic receptor agonist (isoproterenol; ISO) were administered intraperitoneally (i.p.) on a daily basis. White and black bars indicate the light and dark periods, respectively. ZT, zeitgeber time. (**b**–**d**) Peak phase (**b**, **c**) and phase change values (**d**) of PER2::LUC bioluminescence in the submandibular gland in each condition. Phase advance values were calculated as the difference between the peak times of each drug group and the saline group. The number of mice used is indicated in Supplementary Table S1. (**e**, **f**) mRNA expression rhythms (**e**) and total daily mRNA expression (**f**, average of 4 time points) for each adrenergic receptor subtype in the submandibular gland (*n*=6). (**g**) Tissue contents of NE and its metabolite 3-methoxy-4-hydroxyphenylglycol (MHPG), and MHPG/NE ratios in the submandibular gland at ZT0, 6, 12, and 18 (*n*=4). Values are expressed as the mean+/±s.e.m. *P<0.05, **P<0.01, ***P<0.001 versus the young group or the saline group (Student’s *t*-test, Mann–Whitney test, or one-way analysis of variance (ANOVA) with Dunnett’s *post hoc* tests). (**h**) Model of age-related changes in the regulation of peripheral circadian clocks.

Activation of the sympathetic nervous system by stress/exercise is an important signaling pathway for peripheral clock phase adaptation.^32,33^ To examine whether sympathetic peripheral clock regulation is altered in aged mice, we examined phase entrainment of PER2::LUC rhythms in the submandibular gland in response to daily injections of norepinephrine (NE), an α-adrenergic receptor agonist (phenylephrine; PHE), or a β-adrenergic receptor agonist (isoproterenol; ISO) according to a previously reported method.^32^ Dose-dependent phase advancements were observed in response to NE treatment in both young and aged mice, but these shifts were smaller in aged mice than in young mice (Figure 6a,b). Similarly, phase advancements in response to PHE and ISO treatment were smaller in aged mice than in young mice, and this difference was significant in the ISO experiment (Figure 6c,d). We also examined the mRNA expression of each adrenergic receptor subtype in the submandibular gland of intact mice at four different time points during the day (Figure 6e,f; Supplementary Table S3) and found significant decreases in the total daily expression of adrenergic receptors (α1a, α1d, α2c, β1, and β3) in aged mice compared with young mice. These data suggest that decreased adrenergic receptor density contributes to abnormalities in the sympathetic regulation of the submandibular gland peripheral clock in aged mice.

We also examined the abundance of catecholamines including 3-methoxy-4-hydroxyphenylglycol (MHPG) and NE in the submandibular gland. MHPG and the MHPG/NE ratio showed clear circadian rhythms in young mice but not in aged mice (Figure 6g; Supplementary Table S3). These results suggest that impaired daily regulation of the sympathetic nervous system, possibly reflecting the impaired communication of the SCN with peripheral clocks, is involved in age-related differences in circadian entrainment.

## Discussion

The present study led to four major findings (Figure 6h). First, we found that the peripheral clocks of aged mice as evaluated by *in vivo* whole-body imaging of PER2::LUC bioluminescence showed normal rhythmicity in light–dark, constant darkness, and experimental jet lag conditions. Second, the response of aged peripheral clocks to feeding cues was synchronous in all tissues, whereas asynchronous entrainment was observed in young mice (i.e., no entrainment was observed in the submandibular gland). Third, peripheral clock input from the sympathetic nervous system (presumed to originate from the SCN) was attenuated in aged mice relative to young mice. Fourth, the entrainment response of aged peripheral clocks to stress/exercise stimuli was weak, and associated with impaired noradrenergic function and low adrenergic receptor expression. This study is the first to address age-related differences in circadian entrainment using non-invasive *in vivo* bioluminescence monitoring. These results suggest that meal timing is a stronger cue for peripheral clock entrainment in aged animals than in young animals.

In the present study, we hypothesized that aged peripheral clocks would become disorganized when light information was abolished or experimentally shifted due to alterations in behavioral rhythmicity and SCN function.^13,14,16^ Indeed, we observed decreases in the rhythmicity of locomotor activity in the constant darkness condition and age-related differences in the speed of phase changes in locomotor activity during experimental jet lag. However, only small age-related differences in peripheral PER2::LUC bioluminescence were observed under different experimental conditions, and furthermore bioluminescence patterns were always rhythmic and with normal amplitudes. One possible explanation for this observation is that peripheral clock responses to alterations in the light–dark cycle are slower than that of the SCN clock.^21,34^ Alternatively, Sellix *et al.*^21^ reported that peripheral PER2::LUC rhythms in the lung, oesophagus, and thymus showed slower entrainment to a shifted light–dark cycle in aged mice than in young mice, however this study used an *ex vivo* luciferase assay. In partial agreement with this study, we observed slower light entrainment in the submandibular gland of aged mice versus young mice. In contrast, using different conditions, Davidson *et al.*^17^ reported slower entrainment of the hepatic clock (*Per1* expression in cultured tissues) in aged rats compared to young rats. We found that light-induced phase advances in aged peripheral clocks were normal 5 days after the light–dark cycle shift, despite slower entrainment of the activity rhythm. Successful phase advances in aged mice might have been caused by light-induced acute gene expression in peripheral tissues^35,36^ in the absence of a complete shift in the SCN or activity rhythms. Taking together, aged peripheral clocks appear to maintain their amplitudes in different lighting conditions, whereas age-related declines in light-induced entrainment are tissue-specific. Due to a limited number of time samplings in the current study (5 and 9 days after the light–dark cycle shift) and in other studies, additional techniques such as the continuous monitoring of clock gene-driven bioluminescence^37^ are needed to investigate age-related circadian clock changes.

Interestingly, we also found that the fasting-feeding cycle was more important than the light–dark cycle for peripheral clock entrainment in aged mice, as demonstrated by the constant routine feeding experiment. Moreover, feeding behaviors might be remained rhythmic even in the constant darkness condition in the present study. This result is consistent with previous findings indicating that feeding cues are more dominant than light cues for peripheral clock adjustment.^38,39^ Thus, we concluded that the maintenance of peripheral clocks in varied lighting conditions was due to the consistency of fasting-feeding cues.

The function of the sympathetic nervous system in peripheral clock maintenance was highlighted by our data. The present results suggest that the dysregulation of peripheral clocks in aged mice was due to attenuated sympathetic pathway signaling. There are three main evidences in support of this hypothesis. First, in the present study, food-induced entrainment was observed in the submandibular gland of aged mice but not in that of young mice. Sato *et al.*^29^ showed that insulin-insensitive organs including the submandibular gland are more slowly entrained by daytime scheduled feeding than insulin-sensitive organs. In addition, Vujovic *et al.*^30^ reported that *Per1* expression rhythms in the submandibular gland entrained to daytime-scheduled feeding in rats were consistent with sympathetic denervation. Thus, it can be hypothesized that a state of sympathetic denervation was present in aged mice in our study. Second, we observed an increase in food anticipatory activity in aged mice. Walcott and Tate^40^ also noted the appearance of food anticipation in response to scheduled feeding in aged arrhythmic rats. The central clock in the SCN initially responds to light cues and functions to subsequently inhibit food anticipatory activity.^41^ Thus, the present results suggest that age-related impairments in SCN output result in the exaggeration of food anticipation behavior. Third, we observed reduced adrenergic receptor mRNA expression and abnormal catecholamine content in the aged submandibular gland that was associated with peripheral clock arrhythmicity. The observed reduction in adrenergic receptors is consistent with previous studies examining β-adrenergic receptor density and its signal transduction in the aged submandibular gland, brain, and heart.^42–45^ In addition, our data revealed that, among adrenergic receptors, β-adrenergic receptor activation was mainly involved in the entrainment of peripheral clocks in aged mice. Taken together, we posit that age-related impairments in sympathetic function interrupt the internal and external synchrony of the circadian clock system and consequently enhance the dependence of peripheral clocks on feeding cues.

This study provided evidence that, contrary to the effect of aging on scheduled feeding-induced peripheral clock entrainment, entrainment in response to external stress/exercise is impaired in elderly mice. This effect may have been due to age-related decline of the hypothalamic-pituitary-adrenal (HPA) and sympathetic-adrenal-medullary (SAM) axes. Previous studies have demonstrated that HPA and SAM activation have a resetting effect on the circadian clock *in vitro* and *in vivo*.^32,46,47^ In addition, treadmill-induced entrainment of peripheral clocks is dependent on both HPA and SAM axes.^33^ In the present results, we observed a decrease in the response of aged peripheral clocks to NE or DEX in tandem with decreased adrenergic and glucocorticoid receptor mRNA expression in the submandibular gland. Previous studies have identified lower concentrations of baseline and stress-evoked serum corticosterone in aged mice.^48,49^ Alternatively, oxidative stress has been purported to have a role in the stress-induced entrainment of peripheral clocks *in vitro* and *in vivo*.^50,51^ Aging is associated with the accumulation of reactive oxygen species and the impairment of anti-oxidant systems.^5,52,53^ In addition, age-related alterations in anti-oxidant gene expression in the liver has been reported in response to abnormal circadian rhythmicity.^54^ Thus, age-related changes in the HPA and SAM axes as well as the oxidative stress pathway may be involved in decreased entrainment response of peripheral clocks to stress/exercise stimuli in aged mice.

Finally, it is important to note that food-induced entrainment of peripheral clocks was preserved in elderly mice. This effect can be in part attributed to an age-related decline in glucocorticoid function.^48,49^ In a previous study investigating food-induced peripheral clock entrainment, phase changes were accelerated in adrenalectomized and glucocorticoid receptor knockout mice.^55^ Age-related alterations in glucocorticoid signaling were also observed in the present study. Alternatively, our results may suggest that regulated meal timing has an anti-aging effect. Recent evidence indicates that scheduled feeding during the active period helps to reinforce the function of the circadian clock system and can prevent disease progression or age-related changes including diet-induced obesity, cancer development, and cardiovascular dysfunction.^56–59^ Although the improvement of age-related cardiovascular dysfunction by scheduled feeding was demonstrated in a previous *Drosophila* study,^57^ the validation of this effect in higher-level organisms is required in a future study. In humans, daily caloric events are widely dispersed throughout the day, such that regulating the duration of eating events (<10–11 h per day) over a 3-month period was shown to reduce body weight and improve sleep quality in young subjects.^60^ Given that aging in humans is associated with phase advancements and a shortened period in the sleep–wake cycle, as well as decreased sleep duration and fragmented sleep,^11^ further studies are needed to determine whether related meal timings can compensate for the effects of aging on the circadian clock system. The present study thus identifies several fundamental age-related changes in the circadian clock system and provides an important contribution to aging research.

## Materials and methods

### Animals

Visually normal (e.g., without carcinoma or injury) young (3–6 months old) and aged (>18 months old) ICR background heterozygous PER2::LUC knock-in mice^61^ were used in this study. Female mice were used in all experiments except for that demonstrated in Supplementary Figure 1B (male mice). Animals were maintained and used according to the guidelines of the Committee for Animal Experimentation of the School of Science and Engineering at Waseda University and according to the laws of the Japanese government. Mice were maintained on a 12-h light/dark cycle (lights on at 0800 hours) at room temperature (23 °C±0.5 °C) and were provided with a standard MF diet (Oriental Yeast, Tokyo, Japan) and water *ad libitum*. The number of mice used in each experiment is shown in Supplementary Table S1. It should be noted that male and female gendered mice were used according to availability; because aged female mice were considered to be menopausal, the effects of oestrus were only evaluated for young female mice.

### Experimental jet lag

To induce experimental jet lag, mice were placed into individual cages and baseline locomotor activity was monitored using an infrared sensor. After a week of activity recording under the normal light–dark condition, an 8-h light–dark cycle shift was performed by advancing the light period at Zeitgeber time (ZT; ZT0 was defined as the time when the light was switched on and ZT12 was defined as the time when the light was switched off).^16^ Peripheral PER2::LUC rhythms were evaluated 5 or 9 days after the light shift. Imaging was performed starting from ZT15 (previously ZT7).

### Scheduled feeding at ZT4-8

We used a scheduled feeding protocol in which mice were given free access to food only during the feeding period via an automatic feeding apparatus as previously described.^26^

### Constant routine feeding

For the constant feeding routine, mice were placed in individual cages containing automated food dispensers (Pellet Dispenser 45 MG; Med-associates, St Albans, VT, USA). Food pellet meals of equal mass (45 mg per pellet, rodent purified diet, BIO-SERV) were provided on a timed schedule.^31^

### Experimental procedure for the entrainment of peripheral circadian clocks

The ability of peripheral circadian clocks to be entrained by non-photic external stimuli was examined by evaluating the phase advancement of peripheral PER2::LUC rhythms in response to rhythmic stimulation. In a previous study, we found that external stimuli including food, physical/psychological stress, exercise, and adrenergic or glucocorticoid activation presented at regular daily intervals were able to advance or delay the phase of peripheral PER2::LUC rhythms according to stimulation timing.^25^ In this study, we selected ZT4 as the stimulation timing given the fact that phase advancement occurs. For scheduled feeding, mice were fasted overnight, fed 1 g on the first and second days, and fed 1.5 g on the third day. For psychological or physical stress, mice were exposed restraint stress using a wire-mesh bag (3×6×12 cm) or treadmill exercise (12 m/min, Exer-6M Treadmill, Columbus Instrument, OH, USA) for 30 min from ZT4 to ZT4.5 for 3 consecutive days, respectively. For scheduled adrenergic stimulation, NE (1 or 2 mg/kg), ISO (5 mg/kg), PHE (5 mg/kg), or DEX (1 mg/kg) was dissolved in 0.9% saline and administered intraperitoneally at ZT4 for 3 consecutive days. Drugs were purchased from Sigma-Aldrich (St Louis, MO, USA). In all 4 stimulus paradigms, *in vivo* PER2::LUC monitoring was initiated on the third day at ZT7.

### *In vivo* recording of bioluminescence rhythms in peripheral tissues

Bioluminescence oscillations in peripheral tissues were monitored as described previously.^26^ Briefly, mice were anesthetized with a mixture of isoflurane (Mylan, Tokyo, Japan) and concentrated oxygen. D-luciferin potassium salt (Promega, Madison, WI, USA) was injected subcutaneously (15 mg/kg) at the base of the neck between the shoulders. Dorsal and ventral side-up images were acquired with a 1-min exposure time at 8 and 10 min after luciferin injection, respectively, using an *in vivo* imaging system (Perkin Elmer, Waltham, MA, USA). Images were obtained 6 times per day at 4-h intervals using the same mice. Mice were returned to their home cages between imaging sessions. Photon counts for each tissue were analyzed using Living Image 3.2 software (Perkin Elmer). For each individual organ, the average daily photon/sec value was designated as 100% and used to represent daily bioluminescence rhythms. The peak phase, amplitude, and rhythmicity of normalized data were determined using the single cosinor method program (Acro.exe version 3.5).^62^ Cutoff values for rhythmicity (<0.1) were established to determine whether data were rhythmic or arrhythmic, and only rhythmic data were used for analyses of peak phase and average waveforms of normalized PER2::LUC rhythms. Number of tissue samples that met these criteria are shown in Supplementary Table S1.

### Real time RT-PCR

RNA was extracted from peripheral tissues using phenol (Omega Bio-Tek, Norcross, GA, USA). Real-time RT-PCR was performed using the One-Step SYBR RT-PCR Kit (Takara Bio, Shiga, Japan) with specific primer pairs (Supplementary Table S4) and a Piko Real PCR system (Thermo Fisher Scientific, Waltham, MA, USA). Primers were designed using Primer 3 software (produced by Steve Rozen and Helen Skaletsky). The relative expression levels of target genes were normalized to *Gapdh* expression. Data were analyzed using the ΔΔCt method. A melt curve analysis was performed to identify non-specific products. The peak phase and rhythmicity were determined using the Single Cosinor Procedure program, and the results are shown in Supplementary Table S3.

### Activity monitoring

General locomotor activity in their home cage was recorded with an infrared radiation sensor (F5B; Omron, Kyoto, Japan). Double-plotted actograms of locomotor activity are shown with 6 min epochs. Circadian rhythmicity and free-running periods of activity were analyzed by chi-square periodogram analysis with a significance threshold of *P*=0.01 using CLOCKLAB software (Actimetrics, Wilmette, IL, USA).

### High performance liquid chromatography–electrochemical detection (HPLC-ECD)

Tissue monoamine contents (NE and MHPG) were measured by HPLC-ECD (HTEC 500; Eicom, Kyoto, Japan) as described previously.^32^ Data were analyzed using EPC-300 software (Eicom).

### Statistical analysis

Data were analyzed using GraphPad Prism (version 6.03, GraphPad software, San Diego, CA, USA). Equal variance and normal distribution tests were performed to select an appropriate statistical approach for each analysis. Parametric analyses were conducted using a one-way or two-way analysis of variance (ANOVA) with Tukey’s, Dunnett’s, or Student’s *t*-test *post hoc* tests. Non-parametric analyses were conducted using a Kruskal*–*Wallis/Friedman test with Dunn or Mann–Whitney *post hoc* tests. For ANOVA analyses, F and *P* values for each result are shown in Supplementary Table S2. Outliers were identified using a ROUT analysis with *Q*=1%. Data are expressed as the mean±s.e.m. *P*<0.05 was considered to indicate statistical significance.
