# Supplementary material for: Age-related circadian disorganization caused by sympathetic dysfunction in peripheral clock regulation
Source: NPJ Aging Mech Dis. 2017 Jan 5;3:16030–. doi: 10.1038/npjamd.2016.30 (PMC5515066; doi:10.1038/npjamd.2016.30)
Supplement: Supplementary Information [file npjamd201630-s1.pdf]

Supplementary Information

Age-related circadian disorganization caused by sympathetic dysfunction in peripheral clock regulation

Yu Tahara[1, 2], Yuta Takatsu[1], Takuya Shiraishi[1], Yosuke Kikuchi[1], Mayu Yamazaki[1], Hiroaki Motohashi[1], Aya Muto[1], Hiroyuki Sasaki[1], Atsushi Haraguchi[1], Daisuke Kuriki[1], Takahiro J. Nakamura[3], and Shigenobu Shibata*[1]

[1]Laboratory of Physiology and Pharmacology, School of Advanced Science and Engineering, Waseda University, Tokyo, Japan

[2]Waseda Institute for Advanced Study, Waseda University, Tokyo, Japan

[3]Department of Life Sciences, School of Agriculture, Meiji University, Kanagawa, Japan

*Correspondence and proofs should be sent to:
Shigenobu Shibata
Laboratory of Physiology and Pharmacology, School of Advanced Science and Engineering
Waseda University
Wakamatsu-cho 2-2, Shinjuku-ku
Tokyo 162-8480, Japan
Telephone: +81-3-5369-7318; Fax: +81-3-3341-9815; E-mail: shibatas@waseda.jp

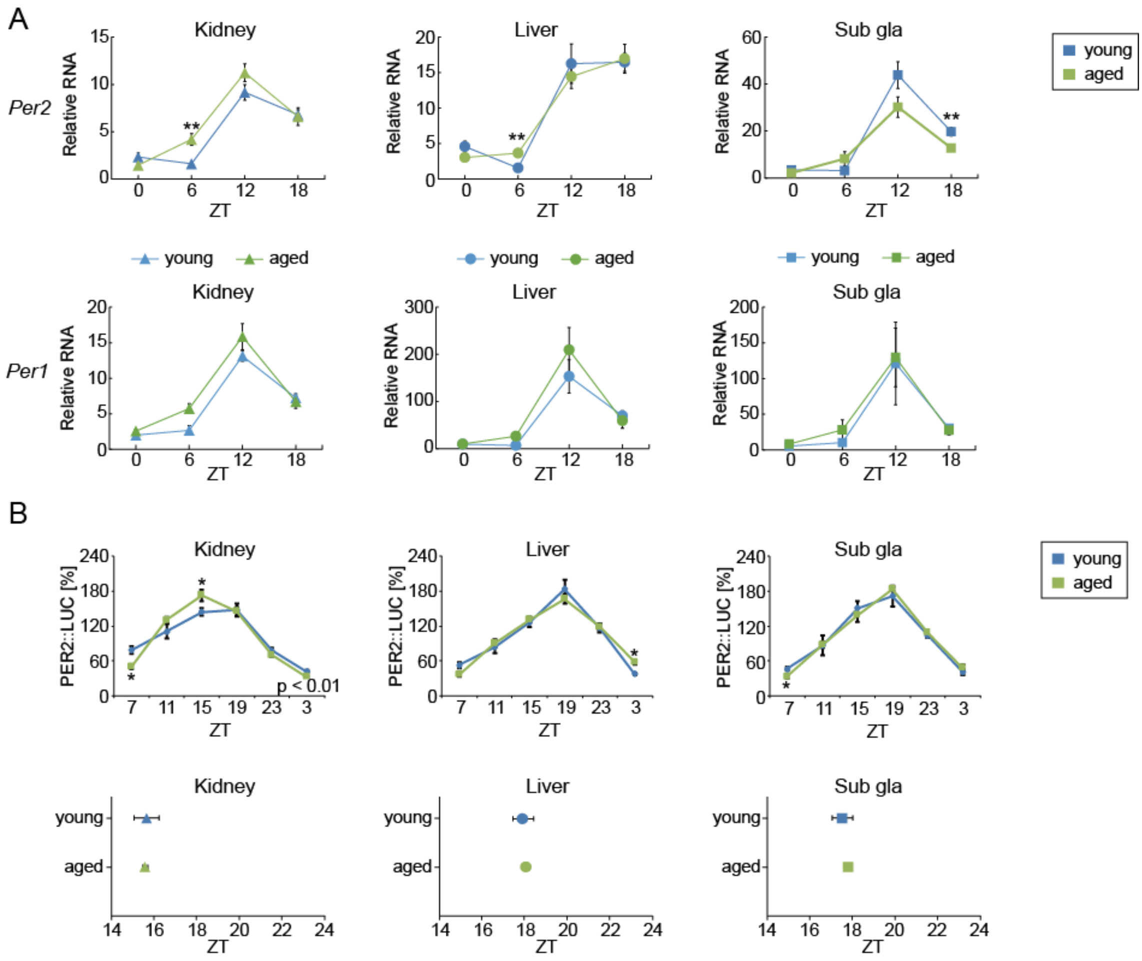

Figure S1. *Per1 and Per2* gene expression rhythms in female mice and peripheral PER2::LUC rhythms in male mice

(A) *Per1* and *Per2* mRNA expression rhythms in the kidney, liver, and submandibular gland (sub gla) of young and aged female mice under the normal light-dark condition (n = 6). ZT, zeitgeber time. (B) Analysed wave forms and peak phases of PER2::LUC bioluminescence in the kidney, liver, and submandibular gland (sub gla) of young and aged male mice under the normal light-dark condition. The number of mice used is indicated in Table S1. Values are expressed as the mean ± SEM. *$P < 0.05$, **$P < 0.01$, vs. the young group (Student's t test, Mann Whitney test, or 2-way ANOVA with Sidak post-hoc tests). The P value on the lower right side of each figure (B) indicates the result of a 2-way ANOVA between the young and aged groups.

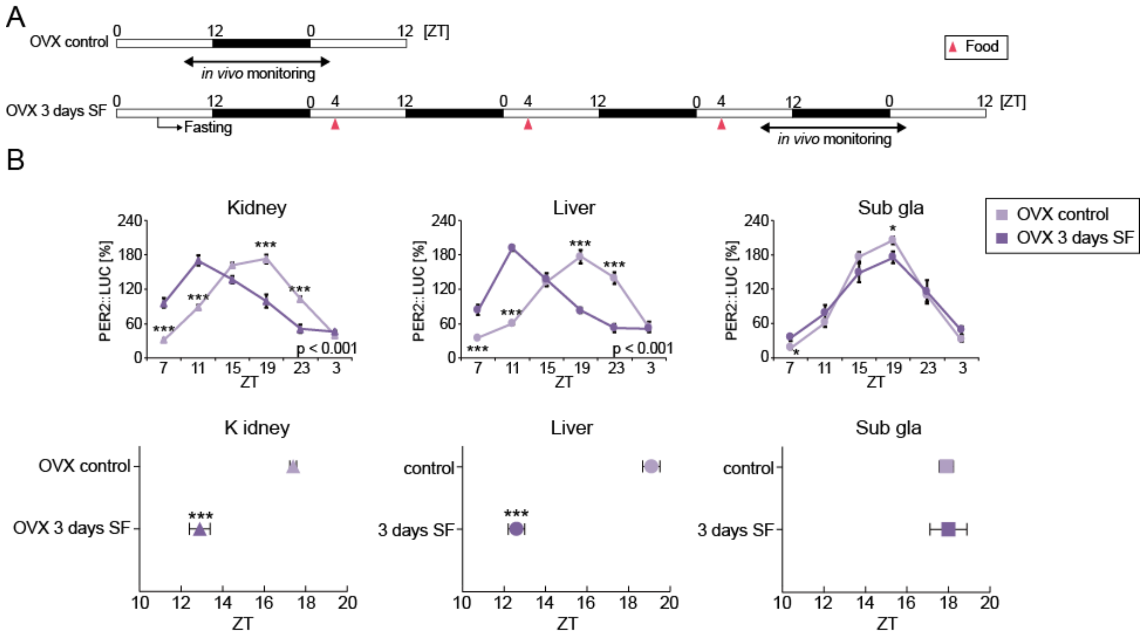

Figure S2. Effect of ovariectomy on peripheral PER2::LUC entrainment to feeding cues

(A) Experimental feeding schedule (SF). Young female mice were ovariectomized (OVX) at least 10 days prior to the onset of the experiment. White and black bars indicate the light and dark periods, respectively. Arrowheads indicate food timings. Food pellets (1 g for the first and second days, 1.5 g for the third day) were given to mice after overnight fasting. ZT, zeitgeber time. (B) Analysed wave forms and peak phases of PER2::LUC bioluminescence in the kidney, liver, and submandibular gland (sub gla). Values are expressed as the mean ± SEM. The number of mice used is indicated in Table S1. *P < 0.05, ***P < 0.001 vs. the OVX control group (Student's t test or 2-way ANOVA with Sidak post-hoc tests). The P value on the lower right side of each figure indicates the result of a 2-way ANOVA between the control and SF groups.

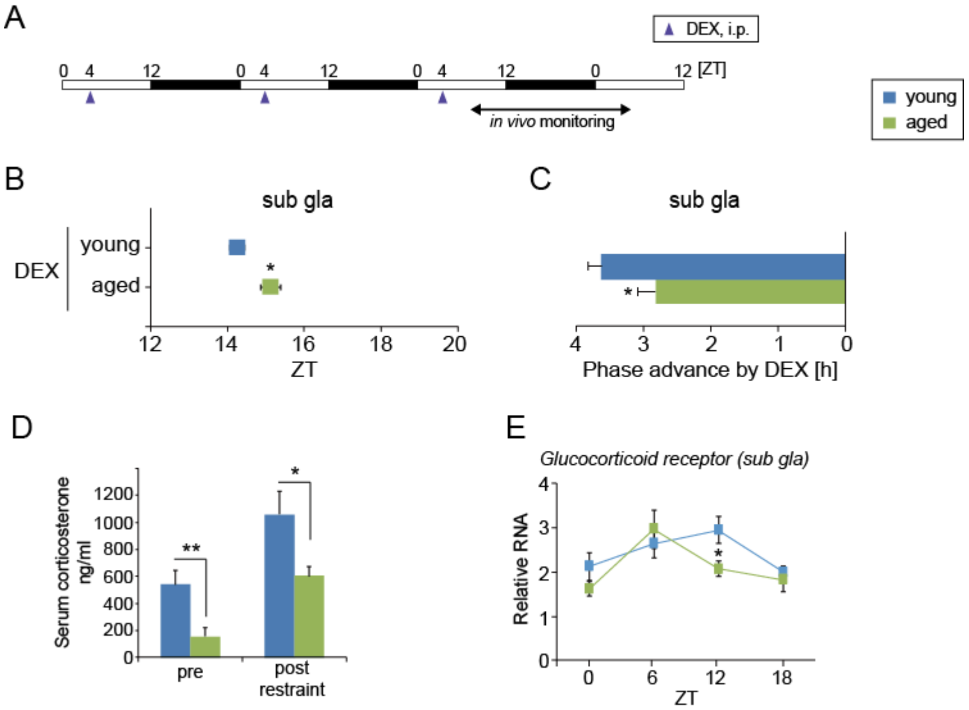

Figure S3. Age-related changes in the glucocorticoid regulation of peripheral clocks

(A) Experimental schedule of glucocorticoid stimulation by intraperitoneal injection of the glucocorticoid analogue dexamethasone (DEX, 1 mg/kg). White and black bars indicate the light and dark periods, respectively. Arrowheads indicate food timings. ZT, zeitgeber time. (B) Peak phases of PER2::LUC rhythms in the submandibular gland after DEX treatment. The number of mice used is indicated in Table S1. (C) Phase advance values of PER2::LUC rhythms in the submandibular gland after DEX treatment. Phase advance values were calculated as the difference between the peak times of the DEX group and the saline group. (D) Serum corticosterone levels at ZT4 (pre) and at ZT4.5 (post restraint stress) on the first day. The restraint stress paradigm was initiated on ZT4 (n = 5–6). (E) mRNA expression rhythms of the glucocorticoid receptor in the submandibular gland (n = 6). *$P < 0.05$, **$P < 0.01$ vs. the young group (Student's t test). Values are expressed as the mean +/± SEM.

Table S1. Numbers of mice examined and tissues that met the rhythmicity criteria in each experimental group.

group name	age	number of mice	number of tissue samples showing rhythmic (rhythmic/total)			Figure No.
			kidney	liver	sub gla	
control, LD, intact, pre	young	6	6/6	6/6	6/6	1, 2, 3, 4, 5
control, LD, intact, pre	aged	9	9/9	9/9	9/9	1, 2, 3, 4, 5
DD	young	4	4/4	4/4	4/4	1
DD	aged	6	6/6	6/6	6/6	1
shift day 5-6	young	10	9/10	7/10	10/10	2
shift day 5-6	aged	8	8/8	7/8	8/8	2
shift day 9-10	young	8	8/8	8/8	8/8	2
shift day 9-10	aged	8	8/8	8/8	8/8	2
2 days SF	young	6	6/6	6/6	6/6	3
3 days SF	young	6	6/6	5/6	6/6	3
2 days SF	aged	8	7/8	6/8	8/8	3
3 days SF	aged	5	5/5	5/5	5/5	3
SF 6 points	young	8	8/8	8/8	8/8	4
SF 6 points	aged	7	7/7	7/7	7/7	4
restraint stress	young	7	7/7	6/7	7/7	5
restraint stress	aged	6	6/6	6/6	6/6	5
treadmill exercise	young	8	8/8	7/8	8/8	5
treadmill exercise	aged	10	10/10	10/10	10/10	5
saline	young	4	4/4	4/4	4/4	6
saline	aged	5	5/5	5/5	5/5	6
NE 1 mg/kg	young	4			4/4	6
NE 1 mg/kg	aged	5			5/5	6
NE 2 mg/kg	young	4			4/4	6
NE 2 mg/kg	aged	5			5/5	6
PHE	young	8			8/8	6
PHE	aged	7			6/7	6
ISO	young	8			8/8	6
ISO	aged	10			10/10	6
male LD	young	3	3/3	3/3	3/3	S1
male LD	aged	4	4/4	4/4	4/4	S1
OVX control	young	4	4/4	4/4	4/4	S2
OVX 3 days SF	young	4	4/4	3/4	4/4	S2
DEX	young	6	6/6	6/6	6/6	S3
DEX	aged	6	6/6	6/6	6/6	S3

Table S2. ANOVA results.

Figure No.	detail	ANOVA	main effect A			main effect B			Interaction effect	
			factor	F	P-value	factor	F	P-value	F	P-value
1E	LD kidney	two-way repeated	time	F (5, 65) = 85.11	p < 0.001	age	F (1, 13) = 1.56	p = 0.23	F (5, 65) = 2.53	p < 0.05
1E	LD liver	two-way repeated	time	F (5, 65) = 61.22	p < 0.001	age	F (1, 13) = 1.56	p = 0.23	F (5, 65) = 1.39	p = 0.23
1E	LD sub gla	two-way repeated	time	F (5, 65) = 85.11	p < 0.001	age	F (1, 13) = 1.56	p = 0.23	F (5, 65) = 2.53	p < 0.05
1E	DD kidney	two-way repeated	time	F (5, 40) = 21.07	p < 0.001	age	F (1, 8) = -12.31	p > 0.99	F (5, 40) = 10.94	p < 0.001
1E	DD liver	two-way repeated	time	F (5, 40) = 21.17	p < 0.001	age	F (1, 8) = 0.00	p > 0.99	F (5, 40) = 21.17	p < 0.001
1E	DD sub gla	two-way repeated	time	F (5, 40) = 16.97	p < 0.001	age	F (1, 8) = 4.57	p = 0.06	F (5, 40) = 8.37	p < 0.001
2C	shift day 5-6 kidney	two-way repeated	time	F (5, 70) = 74.90	p < 0.001	age	F (1, 14) = 26.78	p < 0.001	F (5, 70) = 0.27	p = 0.92
2C	shift day 5-6 liver	two-way repeated	time	F (5, 60) = 43.32	p < 0.001	age	F (1,12) = 4.80	p < 0.05	F (5, 60) = 0.83	p = 0.52
2C	shift day 5-6 sub gla	two-way repeated	time	F (5, 80) = 54.54	p < 0.001	age	F (1, 16) = 19.43	p < 0.001	F (5, 80) = 1.86	p = 0.11
2C	shift day 9-10 kidney	two-way repeated	time	F (5, 70) = 91.86	p < 0.001	age	F (1,14) = 0.87	p = 0.36	F (5, 70) = 4.15	p < 0.01
2C	shift day 9-10 liver	two-way repeated	time	F (5, 70) = 84.01	p < 0.001	age	F (1, 14) = 0.00	p > 0.99	F (5, 70) = 3.19	p < 0.05
2C	shift day 9-10 sub gla	two-way repeated	time	F (5, 70) = 100.50	p < 0.001	age	F (1,14) = 0.00	p > 0.99	F (5, 70) = 1.03	p = 0.40
3E	3 days SF kidney	two-way repeated	time	F (5, 45) = 43.41	p < 0.001	age	F (1, 9) = -7.71	p > 0.99	F (5, 45) = 1.84	p = 0.12
3E	3 days SF liver	two-way repeated	time	F (5, 45) = 48.80	p < 0.001	age	F (1, 9) = -4.80	p > 0.99	F (5, 45) = 1.64	p = 0.16
3E	3 days SF sub gla	two-way repeated	time	F (5, 45) = 16.24	p < 0.001	age	F (1, 9) = 0.00	p > 0.99	F (5, 45) = 5.24	p < 0.001
4B	SF 6 points	two-way repeated	time	F (5, 65) = 46.78	p < 0.001	age	F (1, 13) = -23.11	p > 0.99	F (5, 65) = 4.06	p < 0.01
4B	SF 6 points	two-way repeated	time	F (5, 65) = 36.90	p < 0.001	age	F (1, 13) = 3.25	p = 0.09	F (5, 65) = 2.04	p = 0.08
4B	SF 6 points	two-way repeated	time	F (5, 65) = 99.22	p < 0.001	age	F (1, 13) = -2.60	p > 0.99	F (5, 65) = 6.10	p < 0.001
5B	kidney young	two-way repeated	time	F (5, 55) = 42.67	p < 0.001	age	F (1, 11) = 1.18	p = 0.29	F (5, 55) = 17.48	p < 0.001
5B	liver young	two-way repeated	time	F (5, 50) = 24.26	p < 0.001	age	F (1,10) = 1.00	p = 0.34	F (5, 50) = 2.93	p < 0.05
5B	sub gla young	two-way repeated	time	F (5, 55) = 55.27	p < 0.001	age	F (1, 11) = 1.18	p = 0.29	F (5, 55) = 16.49	p < 0.001
5B	kidney aged	two-way repeated	time	F (5, 65) = 89.86	p < 0.001	age	F (1, 13) = 6.18	p < 0.05	F (5, 65) = 12.50	p < 0.001
5B	liver aged	two-way repeated	time	F (5, 65) = 74.64	p < 0.001	age	F (1, 13) = 1.30	p = 0.27	F (5, 65) = 5.49	p < 0.001
5B	sub gla aged	two-way repeated	time	F (5, 65) = 106.6	p < 0.001	age	F (1,13) = 6.84	p < 0.05	F (5, 65) = 7.56	p < 0.001
5E	treadmill kidney	two-way repeated	time	F (5, 80) = 72.42	p < 0.001	age	F (1,16) = 1.00	p = 0.33	F (5, 80) = 2.38	p < 0.05
5E	treadmill liver	two-way repeated	time	F (5, 75) = 49.73	p < 0.001	age	F (1, 15) = -2.14	p > 0.99	F (5, 75) = 1.41	p = 0.22
5E	treadmill sub gla	two-way repeated	time	F (5, 80) = 62.71	p < 0.001	age	F (1,16) = 10.00	p < 0.01	F (5, 80) = 2.73	p < 0.05
S1B	LD male kidney	two-way repeated	time	F (5, 25) = 87.13	p < 0.001	age	F (1,5) = 0.50	p = 0.50	F (5, 25) = 4.00	p < 0.01
S1B	LD male liver	two-way repeated	time	F (5, 25) = 78.10	p < 0.001	age	F (1,5) = -2.63	p > 0.99	F (5, 25) = 1.54	p = 0.21
S1B	LD male sub gla	two-way repeated	time	F (5, 25) = 68.04	p < 0.001	age	F (1,5) = 38.89	p = 0.00	F (5, 25) = 0.61	p = 0.68
S2B	OVX kidney	two-way repeated	time	F (5, 30) = 74.53	p < 0.001	age	F (1, 6) = 1.77	p = 0.23	F (5, 30) = 37.64	p < 0.001
S2B	OVX liver	two-way repeated	time	F (5, 30) = 39.57	p < 0.001	age	F (1, 6) = 0.00	p > 0.99	F (5, 30) = 52.95	p < 0.001
S2B	OVX sub gla	two-way repeated	time	F (5, 30) = 64.19	p < 0.001	age	F (1, 6) = 0.00	p > 0.99	F (5, 30) = 1.855	p = 0.13

Table S3. Cosinor analysis of RT-PCR data.

Figure No.	Tissue	Name	young		aged	
			Goodness of fit	Achrophase (hr)	Goodness of fit	Achrophase (hr)
6	sub gla	*Adrα1a*	0.049	12.6	0.018	14.4
6	sub gla	*Adrα1b*	0.364	2.4	0.025	21.6
6	sub gla	*Adrα1d*	0.026	9	0.099	2.4
6	sub gla	*Adrα2a*	0.06	5.4	0.028	19.8
6	sub gla	*Adrα2b*	0.139	0.6	0.263	0.6
6	sub gla	*Adrα2c*	0.043	3	0.017	3
6	sub gla	*Adrβ1*	0.252	6	0.016	6.6
6	sub gla	*Adrβ2*	0.023	9	0.221	9.6
6	sub gla	*Adrβ3*	0.047	4.8	0.011	4.8
6	sub gla	NE	0.249	12	0.044	0.6
6	sub gla	MHPG	0.003	19.8	0.145	0.6
6	sub gla	MHPG/NE	0.031	20.4	0.22	18
S1	kidney	*Per2*	0.022	14.4	0.006	13.2
S1	liver	*Per2*	0.018	15.6	0.019	15
S1	sub gla	*Per2*	0.043	13.2	0.02	12.6
S1	kidney	*Per1*	0.028	13.2	0.026	12
S1	liver	*Per1*	0.041	13.8	0.052	12.6
S1	sub gla	*Per1*	0.063	12.6	0.055	12
S3	sub gla	*Glucocorticoid receptor*	0.03	9.6	0.089	7.2

Table S4. Primer sequences for RT-PCR analyses.

Gapdh	5'-tggtgaaggtcggtgtgaac-3'
	5'-aatgaaggggtcgttgatgg-3'
Per1	5'-caagtggcaatgagtccaacg-3'
	5'-cgaagtttgagctcccgaagtg-3'
Per2	5'-tgtgtgcttacacgggtgtccta-3'
	5'-acgtttggtttgcgcatgaa-3'
Adrα1a	5'-atgaggagccaggatacgtg-3'
	5'-tctgacttgtcggtcttgagg-3'
Adrα1b	5'-gctacattggggtgcgatac-3'
	5'-tggacaagacccacacactg-3'
Adrα1d	5'-tctccgtaaggctgctcaag-3'
	5'-aaccagcacaggacgaagac-3'
Adrα2a	5'-aggccatcgagtacaacctg-3'
	5'-cagcgcccttcttctctatg-3'
Adrα2b	5'-gccgagcattggagtacaac-3'
	5'-ttgtagatgaggggcggtag-3'
Adrα2c	5'-tgtatggcatctgccgtgag-3'
	5'-actgttgcagtagccgatcc-3'
Adrβ1	5'-gtaacgtgctggtgatcgtg-3'
	5'-gaaaggcaccaccagcaatc-3'
Adrβ2	5'-ataatctccttggcgtgtgc-3'
	5'-gaactcgcaccagaagttgc-3'
Adrβ3	5'-ccctctccaaaactccatcc-3'
	5'-tttccatcttcctcctgcac-3'
Glucocorticoid receptor	5'-aaatgggcaaaggcgatacc-3'
	5'-gggcaaatgccatgagaaac-3'

Supplemental methods

Ovariectomy

Ovariectomy was carried out using the dorsal approach under midazolam/xylazine-induced anaesthesia. Briefly, the skin of the back was shaved and disinfected, and an incision of approximately 1 cm was made above and parallel-to the spinal cord. Small openings were made in the muscle layers to the left and right of the spinal cord and both ovaries were removed from the surrounding fatty tissue. Ovariectomized mice were allowed to recover for a period of at least 10 days prior to experimental use.

Enzyme-linked immunosorbent assay (ELISA)

Whole blood was allowed to clot at room temperature for at least 30 min, and the resultant clots were removed by centrifugation at 3000 rpm at room temperature for 20 min. An ELISA (Assay Pro, MO, USA) was used to measure serum concentrations of corticosterone according to manufacturer instructions.
